# A non-invasive multipoint product temperature measurement for pharmaceutical lyophilization

**DOI:** 10.1038/s41598-022-16073-x

**Published:** 2022-07-14

**Authors:** Xiaofan Jiang, Petr Kazarin, Michael D. Sinanis, Ahmad Darwish, Nithin Raghunathan, Alina Alexeenko, Dimitrios Peroulis

**Affiliations:** 1 Elmore Family School of Electrical and Computer Engineering, Purdue University, West Lafayette, 47907 USA; 2 School of Aeronautics and Astronautics, Purdue University, West Lafayette, IN 47907 USA; 3 Davidson School of Chemical Engineering, Purdue University, West Lafayette, IN 47907 USA; 4 School of Industrial Engineering, Purdue University, West Lafayette, IN 47907 USA; 5 Birck Nanotechnology Center, Purdue University, West Lafayette, IN 47907 USA

**Keywords:** Computational science, Biomedical engineering, Electrical and electronic engineering

## Abstract

Monitoring product temperature during lyophilization is critical, especially during the process development stage, as the final product may be jeopardized if its process temperature exceeds a threshold value. Also, in-situ temperature monitoring of the product gives the capability of creating an optimized closed-loop lyophilization process. While conventional thermocouples can track product temperature, they are invasive, limited to a single-point measurement, and can significantly alter the freezing and drying behavior of the product in the monitored vial. This work has developed a new methodology that combines non-invasive temperature monitoring and comprehensive modeling. It allows the accurate reconstruction of the complete temperature profile of the product inside the vial during the lyophilization process. The proposed methodology is experimentally validated by combining the sensors’ wirelessly collected data with the advanced multiphysics simulations. The flexible wireless multi-point temperature sensing probe is produced using micro-manufacturing techniques and attached outside the vial, allowing for accurate extraction of the product temperature.

## Introduction

Lyophilization, or freeze-drying, is a commonly used and well-established process that is developed to preserve the original structure of heat-sensitive biological and pharmaceutical products (e.g., antibodies, peptides, vaccines) for more manageable long-term storage and shipment. Freeze-drying involves ice removal from a frozen product at low pressure through a sublimation process. According to the “Markets and Markets” report (https://perma.cc/Z34R-6WX2), the global freeze-drying market is expected to reach $7.3 billion by 2025—from $4.9 billion in 2020—at a Compound Annual Growth Rate (CAGR) of 8.2%. As reported in^1^, about 50% of new injectable/infusible drugs approved by the Food and Drug Administration (FDA) in recent years were manufactured in a sterile powder form requiring lyophilizaiton or a similar drying technology.

Typically, the freeze-drying process is divided into three stages or steps: freezing, primary drying, and secondary drying. At the freezing stage, the solution is completely frozen. In the primary drying step, the chamber pressure is lowered, while heat is supplied to the material for the water to sublime. During this stage, most of the water content is sublimated. The secondary drying step aims to remove the bound water. In this phase, the shelf temperature rises higher than in the primary drying phase to break any physicochemical interactions between the water molecules and the frozen material. The product temperature must not exceed a threshold value throughout the process, particularly during the primary drying stage. This threshold value is a characteristic of the specific product being freeze-dried. For amorphous products, it is often related to the glass transition temperature of the dried product. If the threshold temperature is exceeded, the final dried product may collapse, resulting in poor quality attributes such as higher moisture content leading to faster degradation, a longer reconstitution time or an unacceptable appearance.

Accurate process condition monitoring is not only related to the threshold temperature but is also needed to alleviate machine-to-machine and run-to-run process variations. For instance, a vial heat transfer coefficient and resulting temperature profile are sensitive to variations across different freeze dryers and the spatial distribution of vials inside a given freeze dryer. Although such differences may be less significant in laboratory-scale experiments, they can cause considerable complications in production-level machines.

Inserting miniature fine-gauge thermocouples (TCs) inside the solution to be freeze-dried is the standard industry practice today^2^. TCs were inserted into the vial in this work, affecting the product during drying. This is because the thermal distribution inside the product is altered by the relatively high thermal conductivity of the TCs’ metallic wires with respect to glass conductivity. Also, when a TC comes into direct contact with the drying material, it acts as a nucleation site, thus altering the nucleation process. This may lead to a different structure of the frozen cake^3–5^. Indeed, Bosca et al.^6^ pointed out that the effect is negligible for small sensors; nonetheless differences can still be observed in the drying behavior in the vials with and without TCs. Furthermore, it should be indicated that conventional thermocouples measure temperature only in specific points, which do not necessarily represent the entire product volume. This results in correct product temperature measuring only for a part of the primary drying stage^7^. Also, a thermocouple’s position inside a vial strongly affects temperature reading. Demichela et al. mentioned that operational errors in thermocouple positioning could cause non-trivial temperature measurement uncertainties^8^. Despite these problems, conventional TCs are commonly used to estimate parameters of interest that cannot be measured directly, such as position and temperature of the moving front^9,10^.

More advanced approaches have been proposed to monitor the product temperature of individual vials during the freeze-drying process. A non-invasive temperature monitoring method with thin-film thermocouples (TFTCs) printed on the outside wall was proposed by Oddone et al.^11^. A simplified thermal model is used for the deconvolution of the vial wall temperature to the product temperature. In this work, the deconvolution parameters used in the process were identified using the inner temperature measured by a conventional thermocouple; however, such modeling can be biased by the thermocouple presence^12^. Our previous work^13^ proposed a wireless solution based on low-power sensing electronics to measure product temperature. This approach resolves the TC-induced heating concern while allowing for direct product measurement. However, the sensing is invasive and may interfere with the freeze-drying behavior.

There are several non-invasive solutions for temperature measurements reported in the literature. One of them is the optical fiber sensors (OFSs)^14^ which can be fused in the vial bottom and used non-invasively. Another solution is a sputtered thermocouple method^15^ which includes arrays of sensors. It is worth mentioning the temperature remote interrogation system (TEMPRIS)^16^ which works remotely receiving the energy from the electromagnetic field. One of the latest works of Lietta^17^ describe the use of infrared thermography for monitoring a vial freeze-drying process. The main drawbacks of OFSs are the complexity of manipulations needed to fuse the glass fiber in the vial’s bottom and the impossibility of automatic loading, whereas the sputtered thermocouple also involves a complex manufacturing procedure. The infrared thermography method requires the setup of an IR thermal camera inside the freeze-dryer and can only measure the temperature of the vials standing in front of it. The main disadvantage of TEMPRIS is a large size of an invasive probe. Finally, Barresi^18^ described the “smart vial” concept using thermocouple readings from the side of the vial to reconstruct the temperature inside the vial.

The modeling of the freeze-drying process in a vial has been developing over the last three decades. Pikal^19^ and Millman^20^ have been investigating the freeze-drying process in a vial and developed one-dimensional numerical models. Later, Mascarencas^21^ and Sheehan^22^ developed finite element and multidimensional models for primary and secondary drying modeling. In 2011, Koganti^23^ used modeling to build the design space of the primary drying stage of freeze-drying process. Shivkumar^24^ developed a primary drying simulation, design space generation, and process optimization tool. In one of the recent works, Ravnik et al. proposed a 1D numerical model to simulate the heat transfer and vapor diffusion process in a vial^25^ with accurate capturing of temperature levels inside of the drying material. The model demonstrated a reasonably good agreement with experimental results.

This article presents a new virtual thermocouple technology that allows for a non-invasive, fully wireless, robust measurement approach that overcomes the aforementioned main limitations. This technology comprises three main parts: (a) the flexible non-invasive multi-point sensing probes that are externally attached to the vials, (b) the low-power wireless electronics that read and transmit data wirelessly, and (c) the 2D numerical model that translates the temperature profile measured from the vial wall to the actual product temperature in any point during the primary drying process. This study demonstrates that the proposed method can effectively be used for non-invasive real-time monitoring of the drying dynamics and product temperature during the freeze-drying process.

## Methods

The non-invasive wireless tracking system has been designed to monitor a freeze-drying process across the entire batch with near-zero interaction with the actual product. We are achieving it by monitoring temperature at various locations and tracking the sublimation front of the lyophilization process. This method relies on (a) attaching flexible temperature sensing probes to the outside of the vial and (b) using multiphysics simulation to extract the temperature of the product inside the vial.

### Flexible sensing probe design

**Figure 1:**
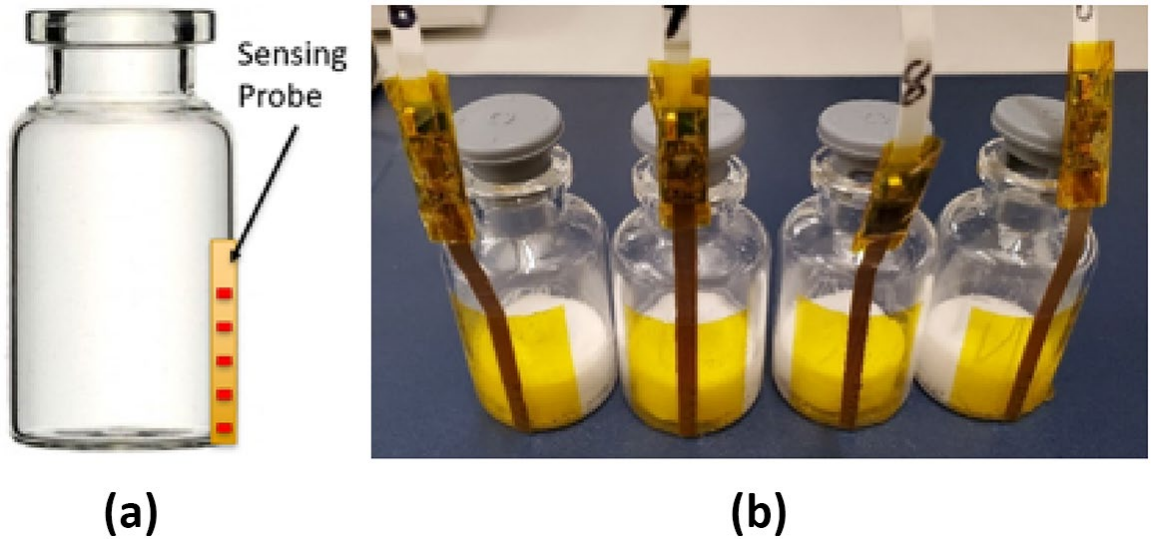
Prototype flexible temperature multi-point sensing probe: (**a**) schematic, and (**b**) real prototype. The sensors are attached to 6R SCHOTT vials whose height and diameter are 40 mm and 22 mm, respectively. Each sensing probe contains 5 sensing elements. The distance between two adjacent sensing elements is 3.05 mm.

A flexible multi-point sensing probe is designed and fabricated using photosensitive lithography and chemical etching. The manufactured device is capable of extracting information concerning the temperature of the product during the lyophilization process. Figure 1 shows a concept and several realized prototypes of the proposed sensor. We use an established, large-scale manufacturing method for standard electronic components to produce the proposed flexible sensor. The sensing device will not require any vial modifications in contrast to Parvis et al.^15^ The wireless and flexible film design allows for use of the sensing element multiple times with different vial sizes and without restricting industrial automatic loading practices. In addition, multiple NTC (Negative Temperature Coefficient) thermistors mounted to the flexible substrate allow measuring temperature at various heights across the vial. The end-user can revise the design accordingly to the vial dimensions used. In this paper, we include five sensing elements in each sensing probe with the bottom element placed at the base of the vial. The distance between two adjacent sensing probes is 3.05 mm.

**Figure 2:**
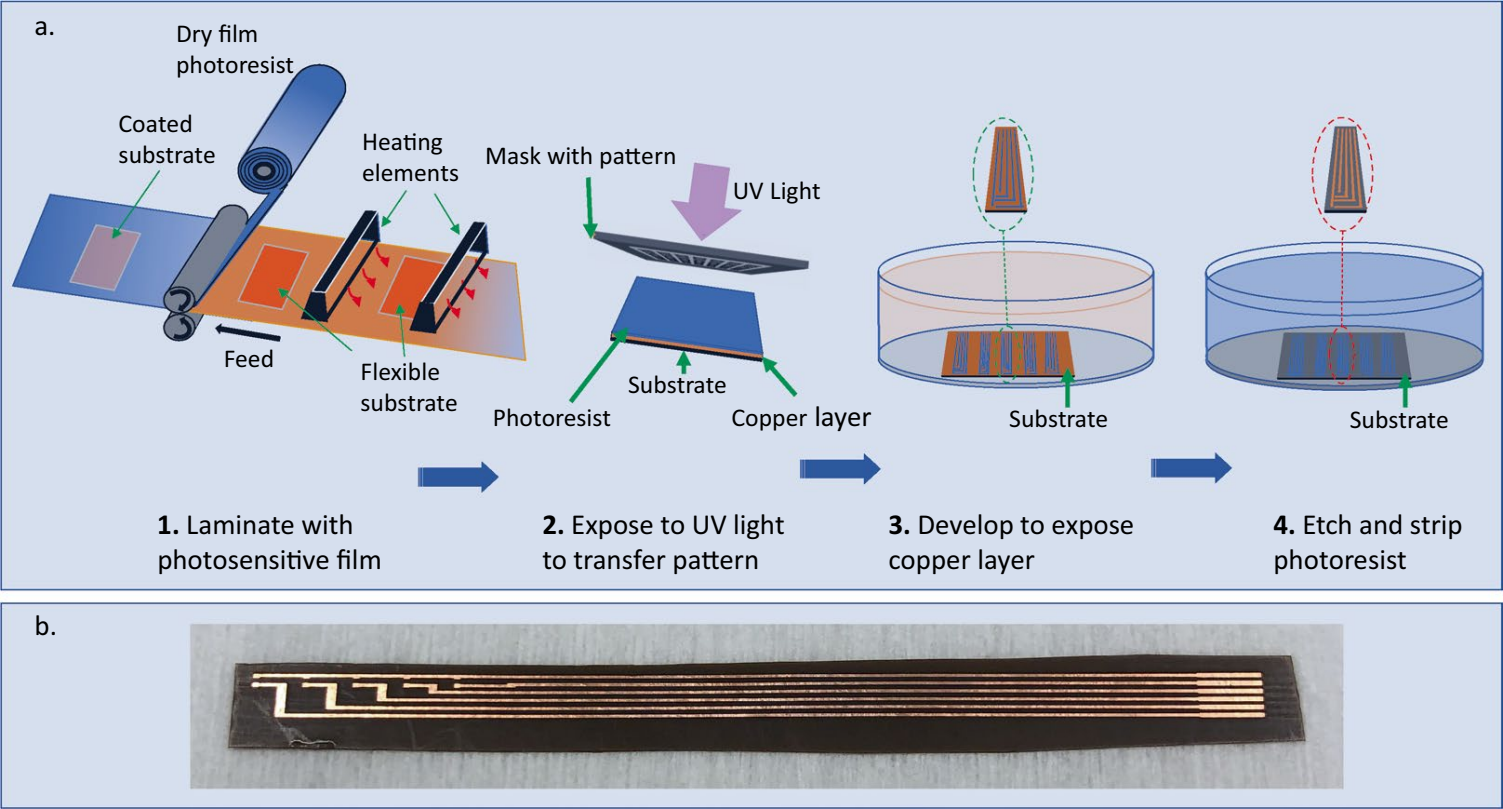
Fabrication process of the flexible Kapton sensors: (**a**) manufacturing steps, (**b**) manufactured sensor substrate.

Figure 2 shows the employed fabrication process for creating the flexible temperature sensors. Sensors are fabricated on copper Kapton laminate Pyralux AP8555R by DuPont. The substrate thickness is 0.127 mm, and the copper thickness is 0.018 mm. The copper is patterned using a photosensitive lithography microfabrication process. Specifically, we used negative dry film photoresist TentMaster TM200i by DuPont hot rolled on the flexible substrate and exposed to 14 mW/cm$^{2}$ of UV light through a photomask using the MA6 Karl Suss aligner. We also used the Copper etchant CE-100 by Transene to form the desired copper traces at the end of the manufacturing step shown in Fig. 2b. The sensor assembly can be transferred on the outside or inside the vial depending on the application, as shown in Fig. 1.

### Temperature sensing element

The NTC thermistor is a small footprint (0.4 mm $\times$ 0.2 mm) electronic component used to sense the product temperature. This thermistor is constructed of metal oxides, which, when passed through a sintering process, give a negative electrical resistance (*R*) dependence versus temperature (*T*). Due to having a large negative slope, a small temperature change will cause a substantial change in electrical resistance at a lower temperature. The disadvantage of such a thermistor is its nonlinear characteristic. Consequently, each thermistor has to be calibrated to ensure measurement accuracy. The Steinhart–Hart (S–H) equation is the most commonly used model to describe the nonlinear characteristic of the thermistor, as shown below.

1 $$\begin{aligned} \frac{1}{T} = A + B~\mathrm{ln}(R) + C~(\mathrm{ln}(R))^{3} \end{aligned}$$

**Figure 3:**
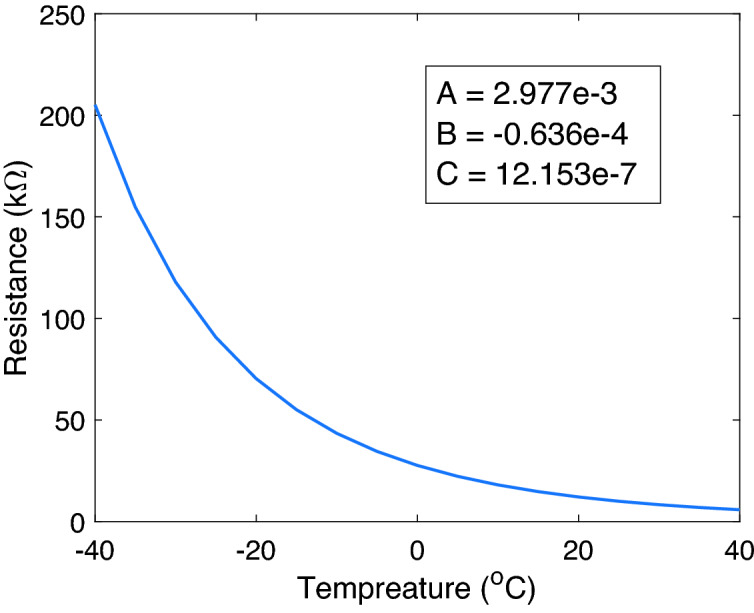
Example of measured and fitted resistance response vs. temperature of the 10K NTC thermocouple.

The symbols are as follows: ﻿*T* is the temperature in degrees Kelvin, ln (*R*) is the natural logarithm of the measured resistance of the thermistor, and *A*, *B*, and *C* are constants. To obtain the values of these constants, each thermistor is used at three different temperatures: 20 $^\circ$C, 0 $^\circ$C, and − 40 $^\circ$C. Subsequently, we fit the coefficients of a third-order polynomial in the log-resistance values to best match the inverse-temperature values (Fig. 3).

### Low-power wireless sensing electronics

**Figure 4:**
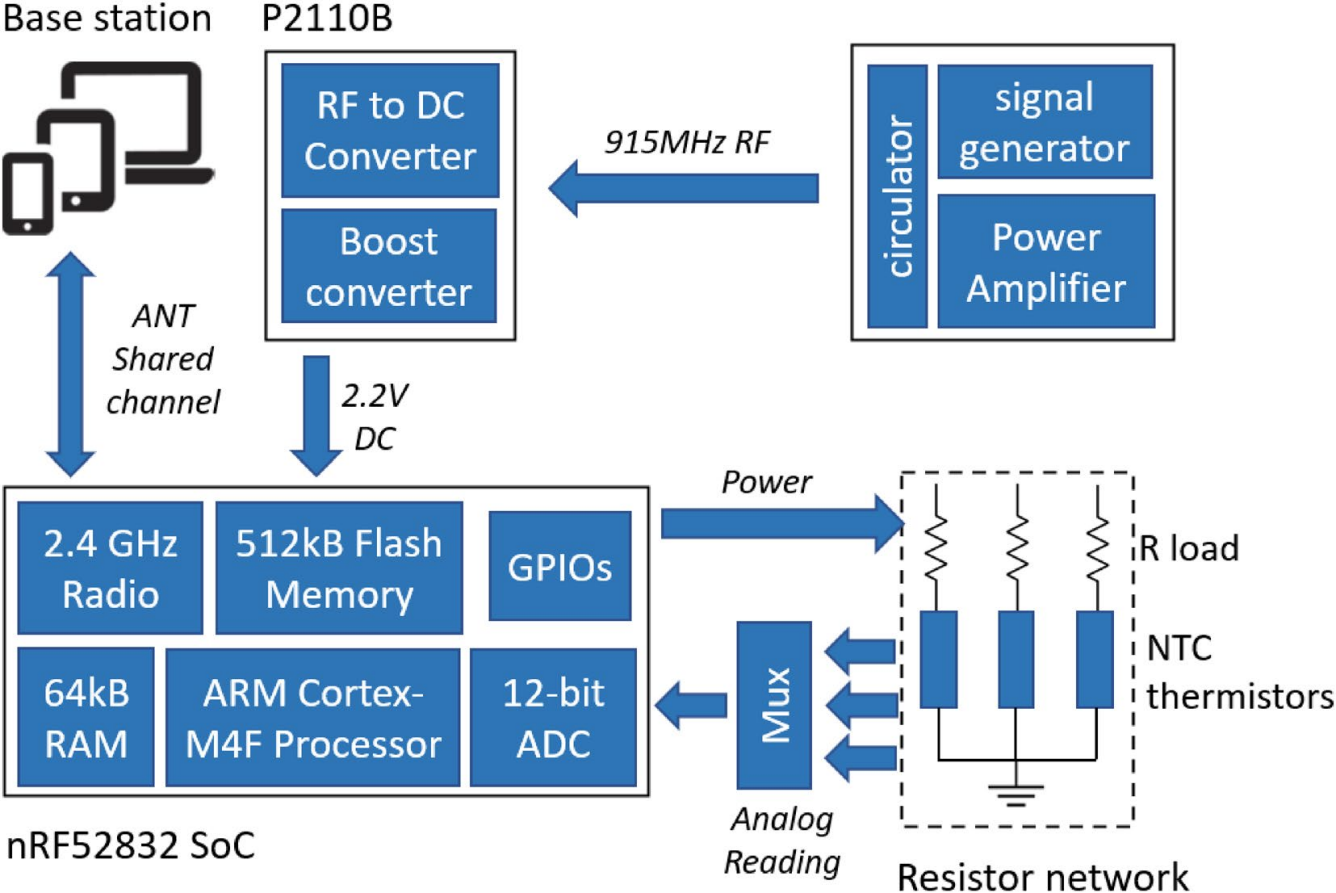
Block diagram of the wireless sensing electronics.

**Figure 5:**
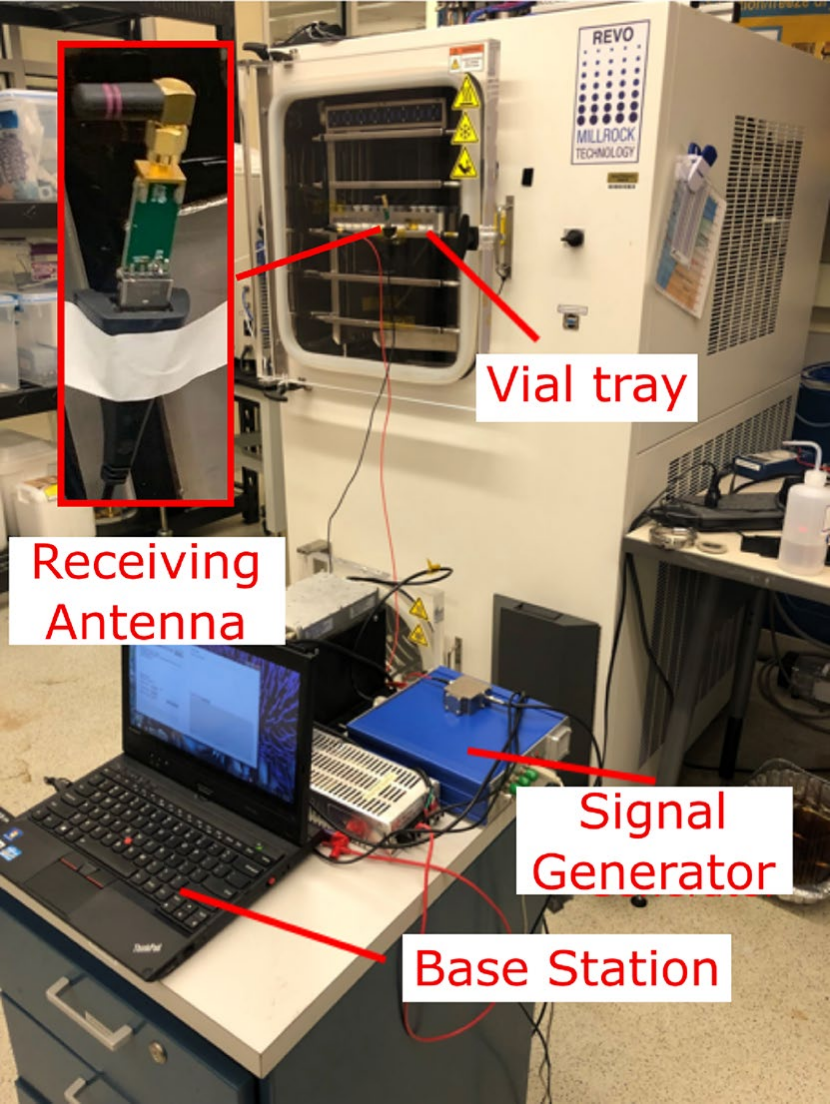
Experimental setup.

Figure 4 shows the block diagram of the low-power wireless sensing electronics. Similar to the previous work^13,26,27^, the nRF52832 system-on-chip from Nordic semiconductor^28^ is employed to process and transmit the measurements via a 2.4 GHz radio link^29^. The transmitting antennas are located inside the chamber on the side of each vial next to the stopper as shown in Fig. 1. The sensing electronics are powered by the P2110B RF harvester from Powercast^30^ which stores the harvested RF energy into a supercapacitor. Temperature sensing also utilizes the build-in 12-bit successive-approximation analog-to-digital converter (SAADC). The temperature sensing thermistors are connected to a $97\,\hbox {k}\Omega$ load resistor. Each voltage dividing circuit is independent for each thermistor and is independently powered by the general-purpose input/output (GPIOs) pins from the micro-controller. The bridge voltage from each voltage-dividing circuit is connected to an 8-to-1 multiplexer, a pre-gain amplifier, and then measured by the built-in 12-bit ADC (0.6 V reference voltage). During operation, the micro-controller dynamically adjusts the pre-gain amplifier for each temperature sensor to counter the nonlinear characteristic of the thermistor and avoid voltage saturation. The 2.4 GHz receiving monopole antenna is located outside of the chamber at the front loading door, as shown in Fig. 5. The system has been successfully tested under typical low pressure and temperature conditions of − 80 C and 50 mTorr. Also, Tipnis et al.^31^ has shown the different methods of electronic sterilization for similar applications.

### Modeling and simulation

To understand the temperature profile measured by the multi-point flexible sensing elements, we create a numerical model for the primary drying stage of the solution in a vial using the COMSOL multiphysics^32^ software. The model allows obtaining the temperature distribution on the vial surface and inside the vial (product temperature). The simulation results are validated against the actual measurements and further investigated.

**Figure 6:**
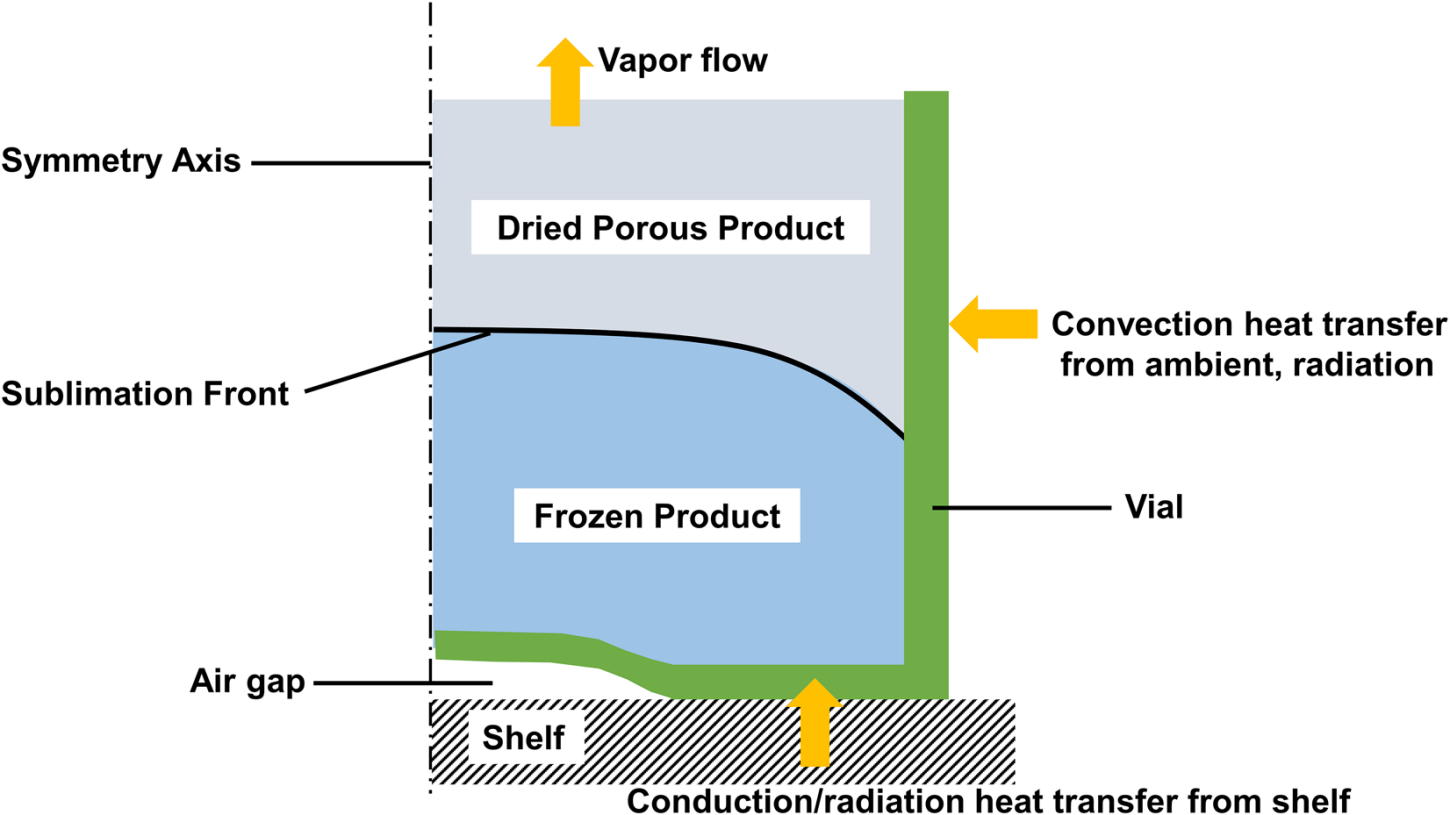
Heat transfer mechanisms between the vial, product, shelf and ambient during the primary drying stage of lyophilization process.

In the proposed model, we numerically solve the transient (time-domain) heat and mass transfer problem during the primary drying stage of the freeze-drying process for the product in a glass vial. The variation of the product and vial temperatures and the position of the sublimation front is predicted. The geometry and the boundary conditions for the 2D axisymmetric problem statement are shown in Fig. 6. The vial is initially filled with frozen $5\%$ mannitol v/v solution. This is split into the frozen zone ($96\%$ of total volume) and the dried zone ($4\%$ of total volume) when the simulation starts.

The following modules were used in COMSOL simulations: “Heat Transfer in Porous Media”, “Darcy’s Law” and “Deformed Geometry”. Several heat-transfer mechanisms are accounted for in this model: convective heat fluxes from ambient, heat exchange between vial, dried/frozen product, and shelf. The heat transfer equations for the ice region without convection and for the dried region with convection are solved. The mass transfer is resolved using Darcy’s law and the vapor density is calculated with the ideal gas law. The heat exchange with the surrounding air and the shelf is represented by the heat transfer coefficients. The dried and frozen regions are assumed to be homogeneous, and the presence of the inert gas during the primary drying process is neglected. The chamber pressure is set at the top of the product. The main parameters’ values used in the simulations are listed in Tables 2, 3 and 4. Fully coupled simulation with multifrontal massively parallel sparse direct (MUMPS) solver with Newton nonlinear method is applied. The temperature at the sublimation interface is calculated using the saturation vapor pressure (Clausius–Clapeyron equation^21,33^):

2 $$\begin{aligned} T_{S} = \frac{2.19\times 10^{-3}L_{S}}{28.89-ln(p)} \end{aligned}$$

where $L_{S}$ is the latent heat of sublimation.

The coupled heat and mass balances are solved on the moving mesh interface and lead to the Stefan condition to obtain the interface velocity:

3 $$\begin{aligned} v_{S} = \frac{Q_{S}}{\varepsilon \rho _{ice}L_{S}} \end{aligned}$$

where $Q_{S}$ is the normal heat flux jump at the interface, $\varepsilon$ is product porosity. This is evaluated using the Lagrange multiplier with enabled weak constraints. Equation (3) describes the Stefan condition for the normal mesh velocity as a part of “Prescribed Normal Mesh Velocity Node”. The ice phase is assumed to be immobile. The transient analysis with the deformed geometry interface is performed to track the ice surface inside the vial (Fig. 6).

**Figure 7:**
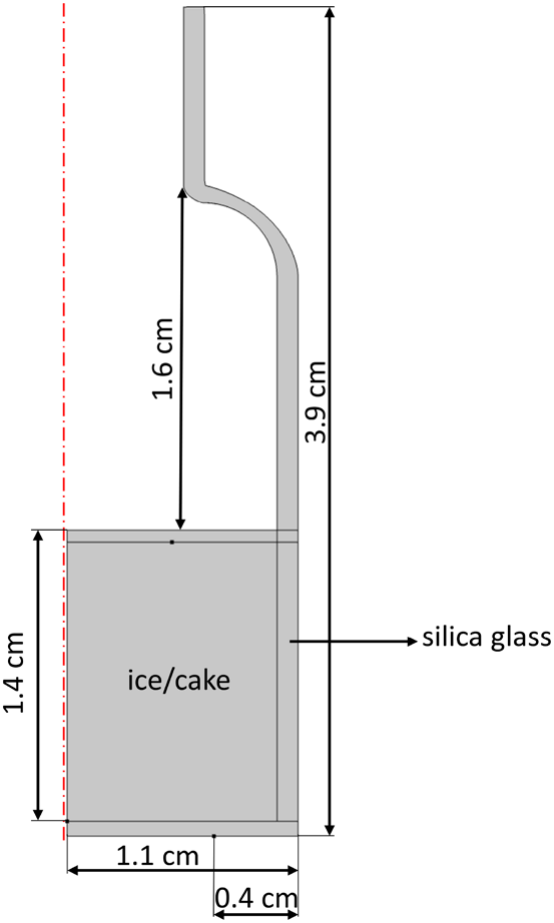
Simplified SCHOTT 6R vial geometry used in simulation.

The simplified vial geometry used in the COMSOL simulation is shown in Fig. 7. Along with the main geometrical dimensions of the vial and materials, the size of the heating zones on the vial bottom are shown: 0.4 cm from the vial edge is used as the zone with higher heat transfer which mimics closer surface-to-surface shelf/vial contact.

### Experimental setup

**Figure 8:**
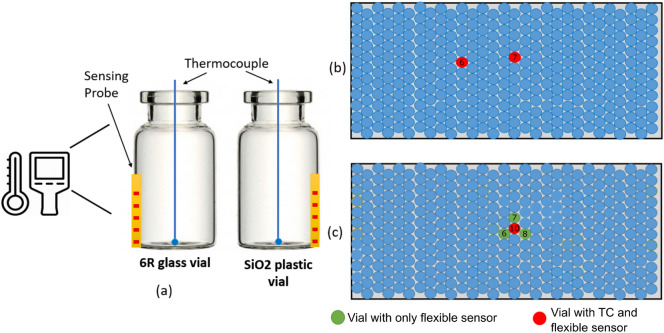
Experimental setups: (**a**) two isolated vials (glass and plastic) with a thermal camera (**b**) 2 center vials equipped with flexible sensors placed in the center of a full tray. (**c**) Experimental set-up for testing the thermocouple heating.

Freeze-drying runs were performed in a laboratory-scale freeze-dryer (REVO, Millrock Technology, Kingston, NY) located at the LyoHub research lab, Purdue University, as shown in Fig. 5. The freeze-dryer is equipped with a vacuum capacitance manometer (MKS Instruments Baratron 622A) and a Pirani gauge pressure sensor (Granville-Phillips 275 Mini-Convectron). A 915-MHz monopole antenna is mounted on the side of the chamber for wirelessly powering the sensors. Also, to prevent leaks and protect the coaxial cable from the vacuum during freeze-drying, a custom vacuum feed-through SMA connector is used to pass the RF coaxial cable inside the chamber to power the antenna. The data-collecting computer is also equipped with a 2.4-GHz ANT-connectivity USB stick for enabling the needed sensor connectivity.

Three sets of freeze-drying experiments are performed with this setup to evaluate the flexible temperature sensor performance. Each set focuses on exploring a different scenario, as described in the following paragraphs. In addition, experiments in each set are repeated at least three times to provide reliable data. Predefined freeze-drying recipes (Table 1) are used in all three runs in 6R SCHOTT pharmaceutical vials with 4 ml filled with 5% D-mannitol solution (Sigma Chemical Company, Germany). Shelf temperature, air temperature, and product temperature were measured with type T conventional thermocouples by Omega on all three experiments.

The first set of experiments (Fig. 8a) focuses on establishing proper sensor performance on two vial types. Specifically, we test the sensors on two different types of vials made of glass (6R SCHOTT vials) and plastic ( SiO$_{2}$ vials: https://www.sio2ms.com/ ). We also insert conventional thermocouples (TCs) at the bottom-center location in each vial type to measure the product temperature. A Thermal IR camera (FLIR Lepton 3.5) is used to monitor the freezing behavior of the product.

The second set of experiments (Fig. 8b) focuses on evaluating the performance of the virtual thermocouple in realistic freeze-drying conditions. In this set, two vials equipped with the flexible sensors, and conventional TCs are placed in the center of the tray. The tray includes a total of approximately 400 vials.

In the third set of experiments (Fig. 8c), four vials equipped with the flexible sensors are placed next to each other in the center of the tray. Unlike the first and second sets, only the center vial (red circle in Fig. 8c) is also equipped with a conventional TC. This set aims to evaluate the conventional TC heating effects with the help of the proposed virtual thermocouple.

**Table 1:** Freeze-drying recipe for 5% w/v mannitol solution in 6R SCHOTT vials.

Freezing step	1	2	3	4
Shelf setpoint ($^\circ \hbox {C}$)	20	20	$-$45	$-$45
Time (min)	0	10	180	120
Primary drying				
Shelf setpoint ($^\circ \hbox {C}$)	$-$45	20	20	
Time (min)	5	60	1800	
Vaccum setpoint (mTorr)	60	60	60	

## Results

### First set of experiments: flexible sensing elements measurements for glass and plastic vials

**Figure 9:**
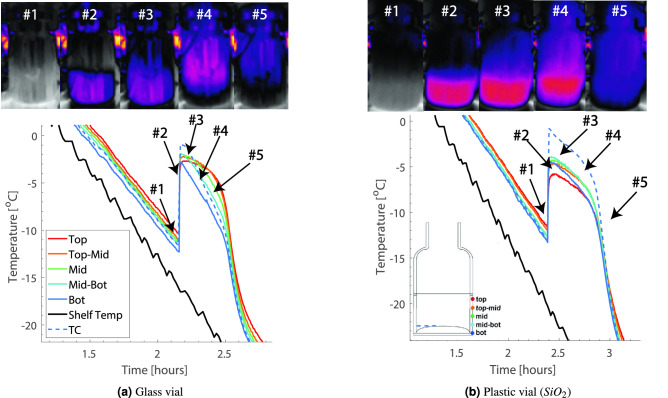
Temperature profile measured by the sensing elements and thermal camera shots (5 moments of time) for the glass and the plastic vials during the freezing stage of 4 ml 5% mannitol solution.

Figure 9a,b show the temperature profile as measured by the five sensing elements of the virtual thermocouple during the freezing stage of the first set of experiments for the glass and plastic vials. In both cases, the bottom sensing element reads the lowest temperature, while the top element shows the highest. This is expected since the bottom sensing element is placed right at the bottom of the vial closest to the shelf. The thermal camera shots for the glass and the plastic vials are also depicted (Fig. 9).

Thermal image #1 shows the moment right before nucleation occurs in both vials. As shown in Fig. 9a,b, uncontrolled nucleation starts right after #1 and results in a sharp rise in temperature (image #2). The thermal camera captures both moments for both vials. However, the two temperature profiles captured by the sensing elements are not identical due to the different thermal conductivity of glass and plastic, different vial thicknesses and masses, and different vial base shapes. All sensing elements quickly rise to − 2 $^\circ$C for the glass vial, just slightly below the product temperature. On the other hand, the flexible sensing elements reach lower temperatures up to − 5 $^\circ$C for the plastic vial.

In addition, the post-nucleation temperature profiles of the two vials are different as well. As the sensing elements indicate on point #4, the glass vial is cooled from the bottom. Temperature is gradually increasing from the bottom to the top of the vial. On the other hand, such a cooling profile was not observed in the case of the plastic vial. The product seems to freeze uniformly inside the plastic vial. These results show that flexible sensing elements successfully capture the freezing dynamics in both vials.

### Second set of experiments: virtual thermocouple performance

#### Multi-physics simulation

We model the primary drying stage and compare the virtual thermocouple readings with actual experimental data. A whole shelf of 6R vials (403 units) filled with 4 ml 5% mannitol solution is freeze-dried in the REVO Millrock lyophilizer. The chamber pressure is set to 60 mTorr and shelf temperature to 20 $^\circ \hbox {C}$. Figure 12 demonstrates the simulated sublimation front position with the vial’s computational mesh and temperature fields and the product for three moments. The porous and solid domains are meshed with a structured mapped grid, while the vial domain meshes with an unstructured grid. The simulation starts with a uniform initial temperature of 228 K for the vial and product, and then the front advances downwards. The automatic re-meshing of the whole geometry occurs when cells’ distortion reaches a certain level. The sublimation stops when the front touches the bottom of the vial after 15.7 h. During the primary drying process, the vial heats the product making the front propagate faster in the vicinity of the vial wall, and it becomes convex. The product and the vial temperatures increase as the simulation advances due to the aforementioned heat transfer mechanisms.

#### Virtual thermocouple measurements

**Figure 10:**
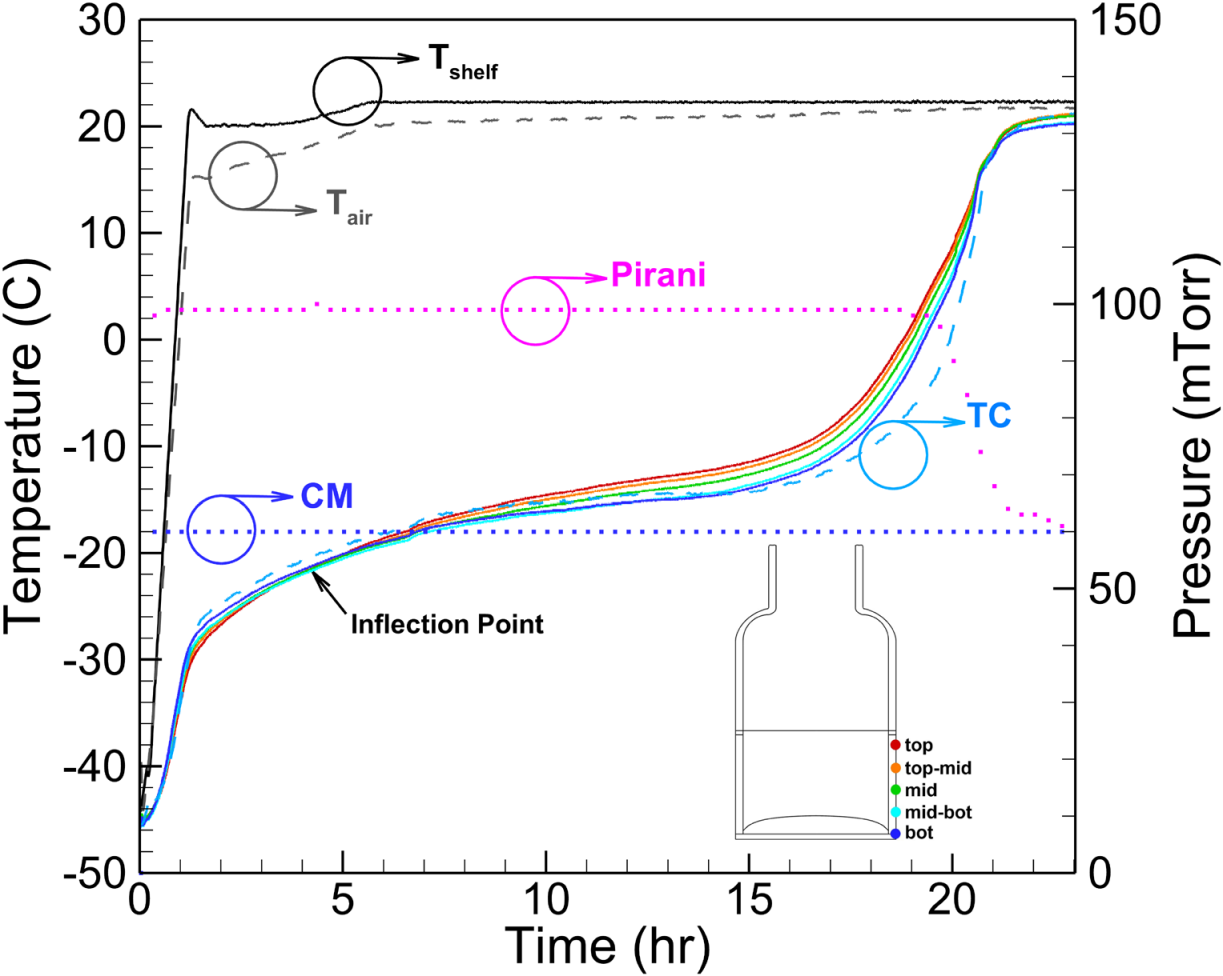
Primary drying stage process parameters for recipe described in Table 1. *CM* capacitance manometer readings and *Pirani* Pirani gauge readings, $T_{sh}$ shelf temperature, $T_{air}$ air temperature in the chamber. Measured product temperature: TC—thermocouple readings and color coded flexible sensor readings of 6R SCHOTT vials filled with 4ml 5% mannitol solution.

Figure 10 illustrates the recorded vial #7 (position in the tray is shown in Fig. 8b) temperature profile during primary drying for a 5% w/v mannitol solution, monitored by two non-invasive flexible sensing elements and two 36 gauge conventional thermocouples placed in the same vials respectively. Also, process data, including shelf temperature, air temperature, and Pirani/capacitance manometer pressure measurement, were also recorded. During this run, the predefined freeze-drying recipes (Table 1) are used with shelf temperature set at 20 $^\circ \hbox {C}$ and a chamber pressure of 60 mTorr. At the beginning of the primary drying, the shelf temperature rises from − 45 to 20 $^\circ \hbox {C}$ . This causes a sharp increase in vial temperature, as observed in both the sensing elements and conventional thermocouple readings. At the beginning of primary drying (after 4 h in Fig. 10), the bottom sensor shows the highest reading as the product temperature rises,and the top sensor shows the lowest. As the primary drying continues and the sublimation front progresses, this trend reverses (inflection point), and the top sensor reading overpasses the top-mid, mid, mid-bot, and bottom sensor ones. As shown in Fig. 10, it is captured by sensing elements readings. The primary drying endpoint can be determined based on the Pirani pressure and capacitance manometer pressure measurements^34^. The primary drying ends as the Pirani reading converges to capacitance manometer measurement. All temperature sensing elements showed perfect agreement in the temperature readings profile with the data obtained from the thermocouples. Interestingly, both multi-point temperature sensing elements indicated an early increase in temperature close to the end of primary drying relative to conventional thermocouple data identifying the vials’ walls heating.

#### Virtual thermocouple and the tuning process

**Figure 11:**
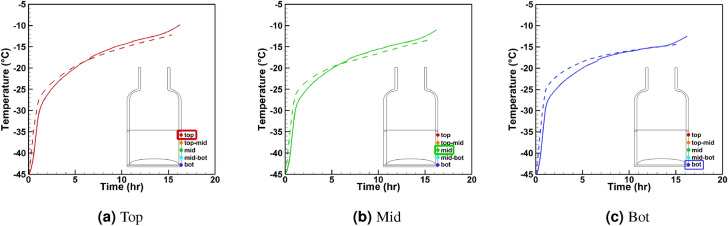
Temperature sensing elements readings vs. virtual thermocouple reading at the vial walls and inside the vial during primary drying stage for three sensors at the center vial #7 (schematic position of the vial is shown in Fig. 8b).

**Figure 12:**
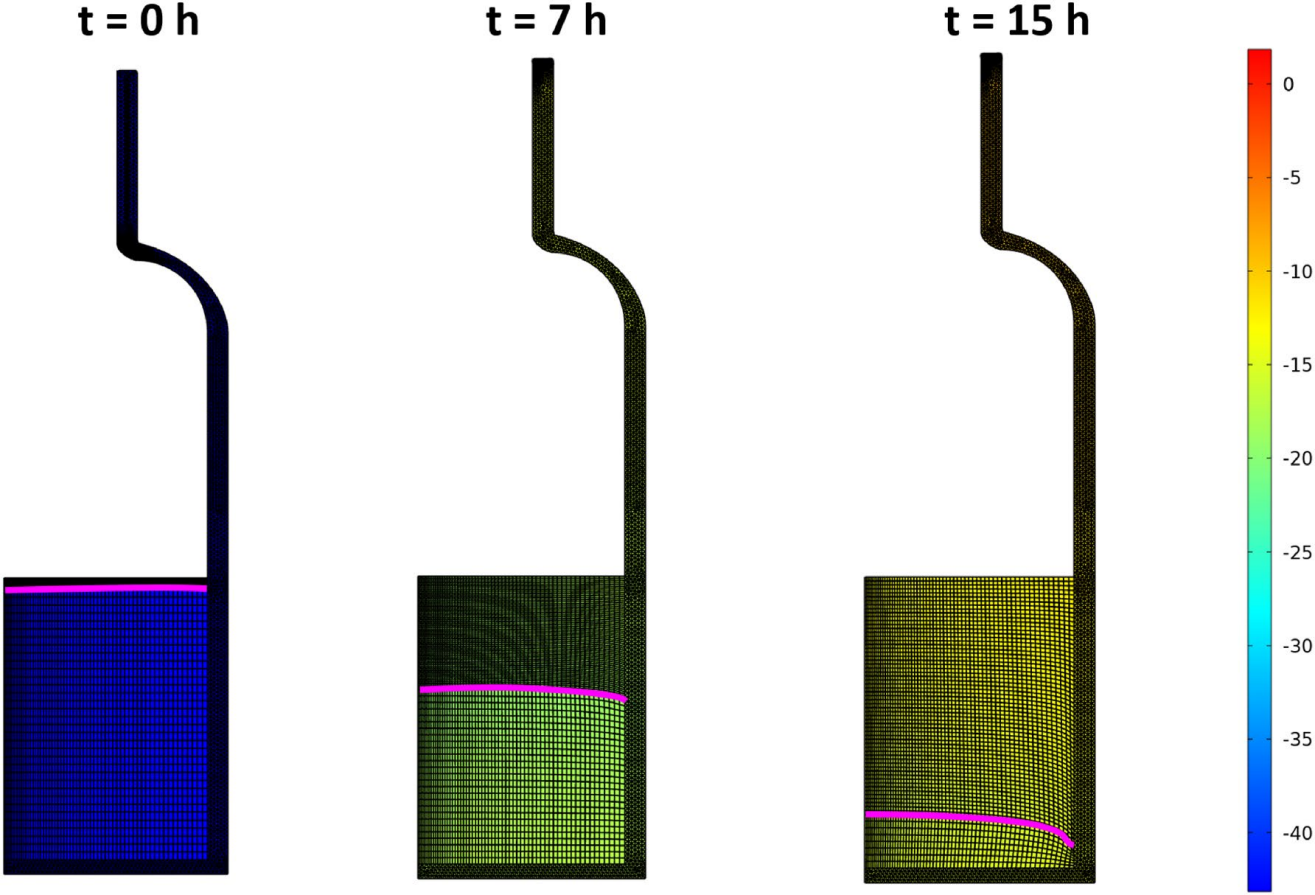
Simulated sublimation front position (purple curve) with computational mesh and temperature fields of the vial and the product for 0, 8 and 15 h.

**Figure 13:**
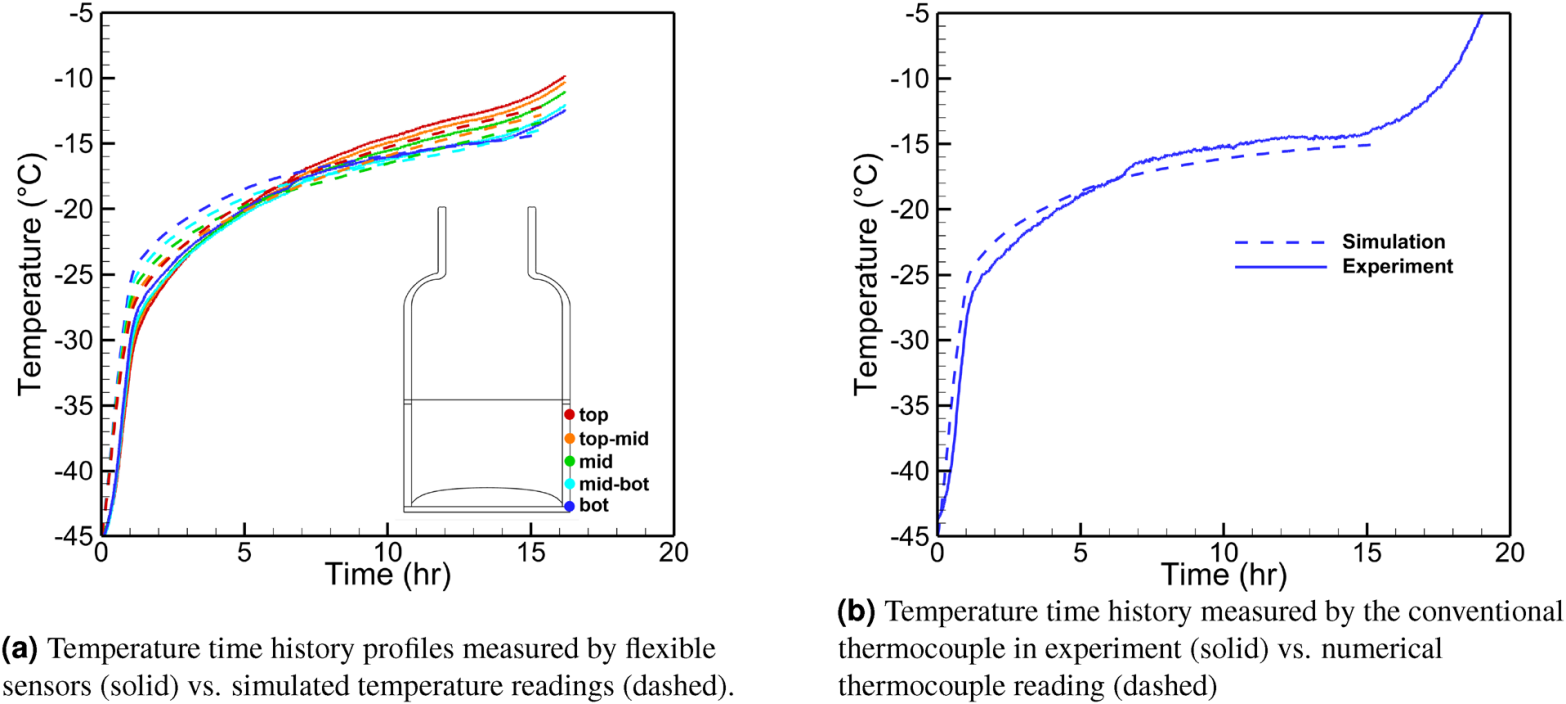
Virtual thermocouple performance evaluation for the central vial #7 (schematic position of the vial is shown in Fig. 8b) during primary drying stage.

**Figure 14:**
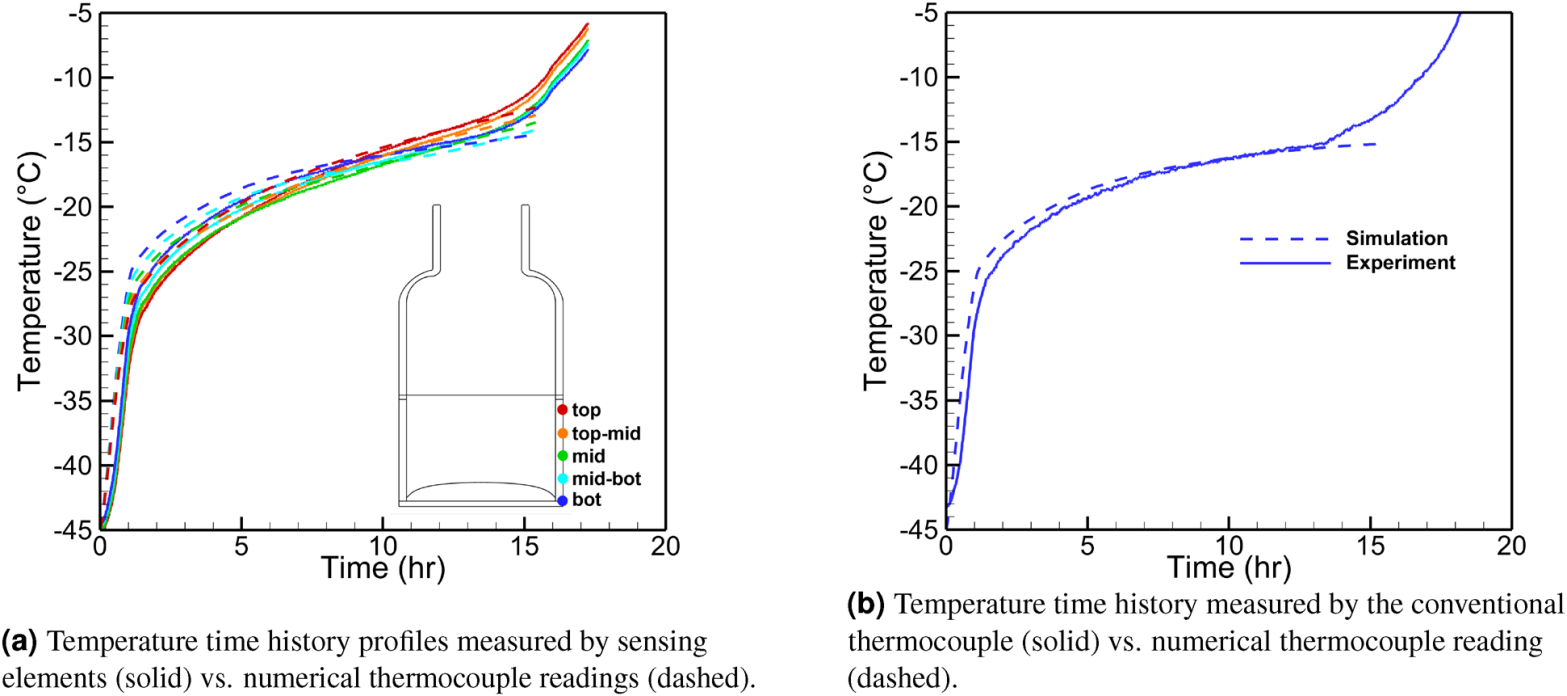
Virtual thermocouple performance evaluation for the central vial #6 (schematic position of the vial is shown in Fig. 8b) during primary drying stage.

The performance of the virtual thermocouple was validated using data from the freeze-drying experiments as mentioned in previous sections. The numerical model was tuned to match the sensing element data during the primary drying stage demonstrated in Fig. 10. As a result, the numerical thermocouple reading should be close to the product temperature measured by a conventional thermocouple in the experiment, which would mean the good performance of virtual thermocouples. Input parameters were divided into three groups: the first is the fixed simulation parameters (Table 2). These parameters are not subject to change from run to run for the same product (such as glass vial properties, material properties (i.e., dried product properties), and ice/vapor characteristics). The second group is the process simulation parameters (Table 3). These parameters are the actual process data, including shelf/air temperatures (measured with conventional thermocouples) and chamber pressure (measured with capacitance manometer) which are directly used in the model. The last group, called the tuned process parameters (Table 4), includes parameters that vary from vial to vial (i.e., heat transfer coefficients). They are tuned to match the virtual thermocouple output with the actual data from sensing elements. The vapor viscosity was calculated using the expression derived by Alexeenko et al.^35^ where the experimentally measured^36–39^ values and the data from The International Association for the Properties of Water and Steam Formulation^40^ were utilized for water vapor viscosity in the range of $-23\;^\circ C$ to $227\;^\circ C$. The power-law curve fit based on Variable Hard Sphere (VHS) molecular model with an effective diameter of $5.78 \text{\AA}$:

4 $$\begin{aligned} \mu =8.9007\times 10^{-6} \left( \frac{T}{273.15}\right) [Pa\times s] \end{aligned}$$

The solid lines in Fig. 11 show the temperature profiles measured by sensing elements. The simulation is performed for vial #6 and vial #7, as indicated in Fig.  8b. Both vials are surrounded by six other vials and can be considered center vials. In both cases, the simulation is within 1 $^\circ \hbox {C}$ of the experiment. The experimental readings of the air temperature in the vicinity of the vial and actual shelf temperature are used in the simulation. Figure  11 shows the measurements from sensing elements versus the virtual thermocouple measurements for vial #6 and 3 sensors: top, middle, and bottom. The close agreement between these readings is demonstrated.

Figures 13b and 14b show the temperature profiles obtained from numerical thermocouple readings and the conventional thermocouple readings after the model is tuned to match the sensing elements data of vials #6 and #7. The heat transfer coefficients tuned to 9 and 12 W/m$^2$/K as well as 8 and 11 W/m$^2$/K for the center and the edge of the vial bottom for two vials, correspondingly. Also, 0.2 W/m$^2$/K heat transfer coefficient was applied to the top part of the vial above the product during the tuning process. The sensing elements temperature readings and simulations results are shown for both vials in Figs.  13a and 14a. The simulation (dashed lines) are within 1$^\circ$–2$^\circ$ from the experimental data during the whole period of primary drying. The deviations close to the end of primary drying are due to the artificial criteria of the end of the process in simulation. The process is assumed to be over when the minimum distance between the freezing front and the vial bottom is close to zero. Thus, when the edge of the sublimation front reaches the bottom of the vial, the simulation stops. As shown in Figs.  13b and 14b, the numerical thermocouple temperature data show an excellent agreement with the conventional thermocouple reading. Thus, the virtual thermocouple is shown to measure the actual product temperature accurately and non-invasively.

**Table 2:** Fixed simulation parameters.

Parameter	Dimension	Value
Ice heat capacity	*J*/*Kg*/*K*	1967.8
Product heat capacity	*J*/*kg*/*K*	1715
Vapor heat capacity	*J*/*kg*/*K*	1674.7
Latent heat of sublimation	*J*/*kg*	$2.838\times 10^{-6}$
Ice thermal conductivity	*W*/*m*/*K*	2.1
Product thermal conductivity	*W*/*m*/*K*	0.028
Vapor thermal conductivity	*W*/*m*/*K*	0.025
Vapor molar mass	*g*/*mol*	18
Vapor viscosity	$Pa\times s$	Equation 4
Ice density	$kg/m^3$	913
Product density	$kg/m^3$	75
Silica glass heat capacity	*J*/*kg*/*K*	830
Silica glass density	$kg/m^3$	2230
Silica glass thermal conductivity	*W*/*m*/*K*	1.14

**Table 3:** Process simulation parameters.

Parameter	Dimension	Value
Air temperature	*K*	Exp.
Initial temperature	*K*	228
Shelf temperature	*K*	Exp.
Chamber pressure	*mTorr*	70

**Table 4:** Tuned simulation parameters.

Parameter	Dimension	Value
Product permeability	$m^2$	$3\times 10^{-6}$
Vial bottom heat transfer coefficient (in/out)	$W/m^2/K$	Variable
Porosity	–	95%

**Figure 15:**
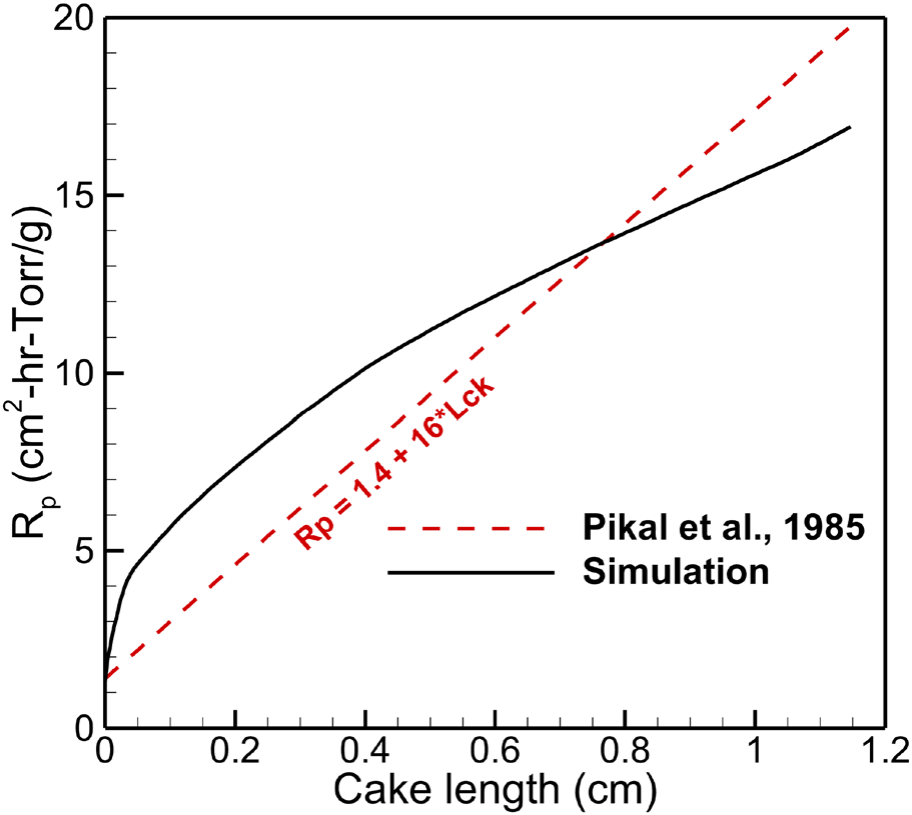
Product resistance calculated based on the simulation of primary drying stage of 4 ml 5% mannitol solution in 6R SCHOTT vials vs $R_{p}$ from Pikal et al.^41^.

Figure 15 shows the mass transfer resistance calculated for a dried cake of 5% mannitol solution and compared with empirically obtained expression by Pikal et al.^41^ as a function of the dried thickness or cake thickness $L_{ck}$ as:

5 $$\begin{aligned} R_{p} = A_0 + \frac{A_{1}\times L_{ck}}{1+A_2\times L_{ck}} \end{aligned}$$

where $$A_0 = 1.4$$, $$A_1 = 16$$, $$A_2 = 0$$.

The cake resistance from the current simulation is calculated according to^19^:

6 $$\begin{aligned} R_{p} = \frac{A_{p}\times (P_{sub}-P_{ch})}{\dot{m}_{ice}} \end{aligned}$$

where $A_{p}$ is a product area, $P_{sub}$ and $P_{ch}$ are sublimation front and chamber pressures, $\dot{m}_{ice}$ is an ice sublimation rate. The $R_p$ measures vapor flow impedance resulting from the dried layer structure. It is worth noting that in the current multiphysics simulation, the product permeability is one of the parameters analogous to $R_p$. From Fig. 15, one can see that $R_p$ curve of 5% mannitol was calculated using the data from COMSOL simulation, and the one obtained by Pikal et al.^41^ are very close. The product’s resistance is a parameter that can be affected by the freezing protocol, particularly with the temperature of nucleation. Even though the current simulation considers the nonuniform heating of the vial and accounts for product and vial properties, some factors cannot be directly measured and accounted for in the simulation. For example, the relative position of the vials on the shelf can vary due to the loading process or human factor, the variation in shelf heat transfer and pressure distribution within the chamber are also among such factors. In general, in addition to the product temperature match, the shape of the $R_p$ curve from the simulation shows that the model closely reflects the physics of the actual process.

### Third set of experiments: conventional thermocouple heating

**Figure 16:**
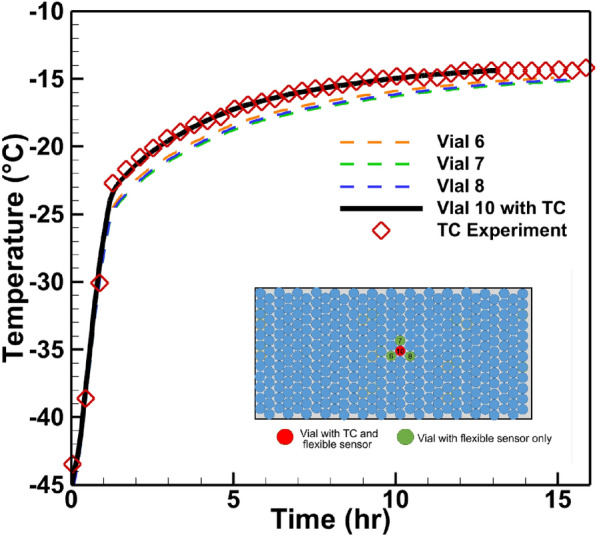
Product temperature profiles during primary drying stage. Virtual thermocouple readings for vials without thermocouple (Vial 6, 7, 8), vial with thermocouple (Vial 10 with TC) and experimental thermocouple readings (TC Experiment).

With the ability to measure the product temperature close to the center of the vial bottom during primary drying, we utilize the power of the virtual thermocouple to investigate the effects of conventional thermocouple heating. Figure 8c shows the setup of this experiment, where three vials equipped with a virtual thermocouple were placed at the center of a full tray (green dots in Fig. 8c), surrounding a vial equipped with both virtual thermocouple as well as conventional thermocouple. This effect is demonstrated in Fig.  16. The temperature at the walls of four vials in the shelf center was measured using sensing elements. For each vial, the simulation was performed, and heat transfer coefficients were adjusted so that the best agreement between experimental sensing elements readings and simulation is achieved. From Fig.  16, it can be seen that in vial #10, a perfect agreement between the conventional thermocouple measurement and numerical thermocouple simulation is reached. The heat transfer coefficient was tuned for other vials to get the experiment/simulation agreement. Figure 16 shows four numerical thermocouples readings in four vials as well as one conventional thermocouple reading in vial #10. There is a perfect match between conventional/numerical thermocouple readings in vial #10. The average difference between the virtual thermocouple product temperature reading and the one registered by conventional thermocouple reading is $1.01\,^\circ$C (with vial 6) and $1.37\,^\circ$C (with vial 7). The maximum difference is $1.56\,^\circ$C (with vial 7). These calculated temperature differences are due to the presence of the conventional thermocouple in a vial. Thus, the use of flexible sensors allows performing actual temperature measurements. Additional experiments demonstrating the effect of thermocouple on the sublimation process are presented in the Supplementary Information.

## Discussion

The development of optimal lyophilization procedures for different formulations in vials includes experimental tests and computational approaches for measuring product temperature. Tight temperature control is essential in both the freezing and primary drying steps because the freezing and primary drying protocols determine the structure of the dried product (cake). To obtain the uniformly dried product across the batch, one needs to control the temperature during these stages accurately. Notably, ice nucleation during the freezing stage should occur in a tight temperature interval. Most importantly, the product temperature must be kept safely below the collapse temperature during the primary drying stage. Due to the presence of bound water in the product after the primary drying stage, the collapse temperature can be relatively low. Moreover, the critical process parameters should be controlled accordingly to optimize the process and reduce the primary stage duration. Along with the chamber pressure, shelf temperature is one of such parameters which defines the design space for the primary drying stage of the freeze-drying process. Traditionally, the shelf temperature depends on the temperature of heat transfer fluid (i.e., silicon oil or methylene chloride) inside the shelves, which is tracked by the control system and is set to follow the pre-set profile. However, the heat transfer control obtained by the control and manipulation of the shelf temperature is relatively slow, partly because of the thermal inertia of the system, due to which shelf heating and cooling may induce a considerable lag in the response of the product temperature. Alternatively, the chamber pressure of the dryer can be controlled and manipulated since the heat flux from shelf to product strongly depends on it. However, this approach can be pretty risky because the product temperature practically follows the pressure variations; therefore, changes of a few pascals could easily jeopardize the product quality.

Since the critical part of any lyophilization procedure is the primary drying stage, special attention has to be paid to the critical modeling parameters of drying a porous cake-solid ice system. In this work, a new technology, virtual thermocouple, based on the use of flexible multi-point temperature sensor and advanced multi-physics simulation, was proposed and investigated as a means for the monitoring of freezing and drying behavior and product temperature during the freeze-drying process. The developed virtual thermocouple combining the two-dimensional model with the surface sublimation sub-model can be used as a stand-alone, fast, and accurate computational tool to predict lyophilization dynamics. However, it can also be included in a general 3D CFD computational framework as a vital part of the final virtual lyophilizer model. Moreover, the two-dimensional freeze-drying model can be extended into three dimensions and accurately capture the non-uniform vial heating effects such as edge effect and 3D shape of the sublimation front inside the vial. The proposed virtual thermocouple was found to give quantitatively accurate results for drying behavior. In particular, the flexible multi-point sensing elements can give information about the temperature profile on the vial wall. This information, combined with the advanced multi-physics simulation, provides the actual product temperature, position, and shape of the sublimating interface and perfectly matches conventional thermocouple measurement. The proposed virtual thermocouple technology can effectively track the temperature profile within the volume of the solution of an individual vial during the freeze-drying process.

## Supplementary Information

Supplementary Information.
