# Supplementary material for: A non-invasive multipoint product temperature measurement for pharmaceutical lyophilization
Source: Sci Rep. 2022 Jul 14;12:12010. doi: 10.1038/s41598-022-16073-x (PMC9283482; doi:10.1038/s41598-022-16073-x)
Supplement: Supplementary file 1 — Supplementary Information. [file 41598_2022_16073_MOESM1_ESM.pdf]

Supplementary Information for "A Non-Invasive Multipoint Product Temperature Measurement for Pharmaceutical Lyophilization"

Xiaofan Jiang[1,+], Petr Kazarin[2,5+], Michael D. Sinanis[1,4,5], Ahmad Darwish[1,5], Nithin Raghunathan[4], Alina Alexeenko[2,3,5,*], and Dimitrios Peroulis[1,5]

[1]Elmore Family School of Electrical and Computer Engineering, Purdue University, West Lafayette, 47907, USA
[2]School of Aeronautics and Astronautics, Purdue University, West Lafayette, IN 47907, USA
[3]Davidson School of Chemical Engineering, Purdue University, West Lafayette, IN 47907, USA
[4]School of Industrial Engineering, Purdue University, West Lafayette, IN 47907, USA
[5]Birck Nanotechnology Center, Purdue University, West Lafayette, IN 47907, USA
[*]alexeenk@purdue.edu
[+]these authors contributed equally to this work

Supporting Information Text

Experiment 1

To demonstrate the effects of the thermocouple (TC) in the sublimation process we conducted the following experiment: A batch of 5%, 4 mL mannitol using SCHOTT 6R Vials was prepared, as depicted in Supplementary Figure 1. The chosen sample for testing was set to twelve vials, six out of which contain conventional TCs. Eight vials were centered and the remaining four were placed at the edge of the shelf. Shelf symmetry was considered when choosing the sample under test to maintain similar thermal conditions for the vials considered. Lyophilization cycle parameters were set at T_{sh} = 20°C and P_{ch} = 60 mTorr. The weights of samples under test were measured before and after the lyophilization cycle, which was stopped 15 hours after the beginning of the primary drying (PD), before the products were fully dried. Supplementary Figure 2 shows that the observed results confirm the impact on the sublimation rate due to the thermocouple presence. By individually comparing every symmetrical pair of vials, the largest difference of 11.2% was observed between vials 4 and 10. On the other hand, the minimum observed difference is 0.74% between vials 2 and 8. The difference in averages is shown with the horizontal lines in Supplementary Figure 2. Based on the observed results of the Histogram, on average, the sublimated water amount is higher when conventional TCs are placed inside the vial, which agrees well with our claim.

Experiment 2

To further confirm the observed results discussed in Experiment 1, we selected the vials around the center of the shelf as depicted in Supplementary Figure 3. The conditions of this experiment were identical to experimental 1 (5%, 4 mL mannitol in SCHOTT 6R Vials; T_{sh} = 20 C; P_{ch} = 60 mTorr). The location was considered having in mind that the shelf center is partially isolated from the shelf edge effects. The obtained results (Supplementary Figure 4) agree well with experiment 1, verifying that when TCs are used, the amount of sublimated water is higher.

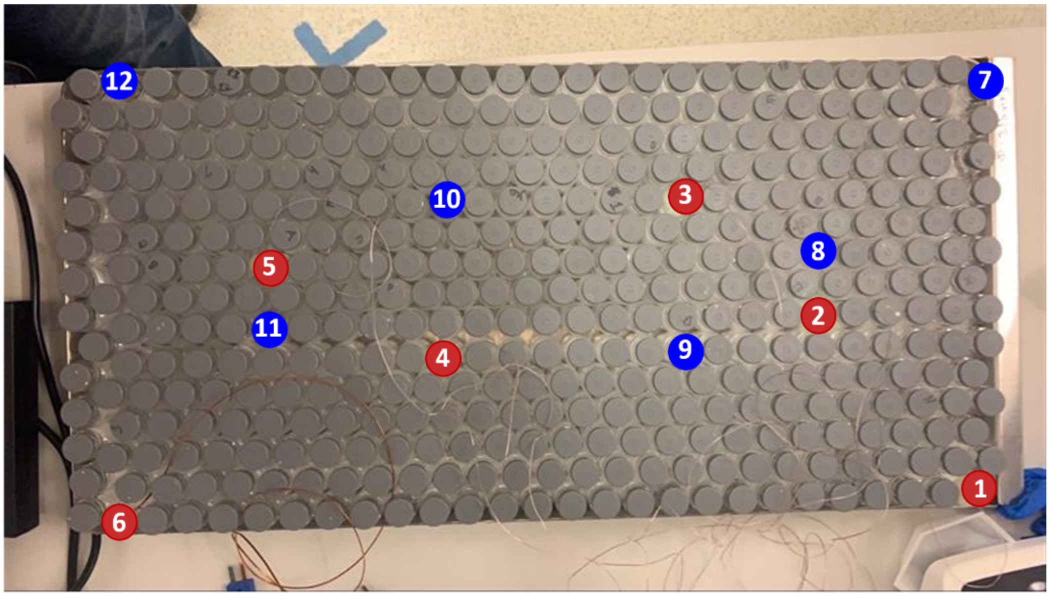

Supplementary Figure 1. The photo of the REVO Millrock shelf with vials: 6R SCHOTT Vials filled with 4 ml 5% mannitol solution. The 12 investigated vials are color-marked.

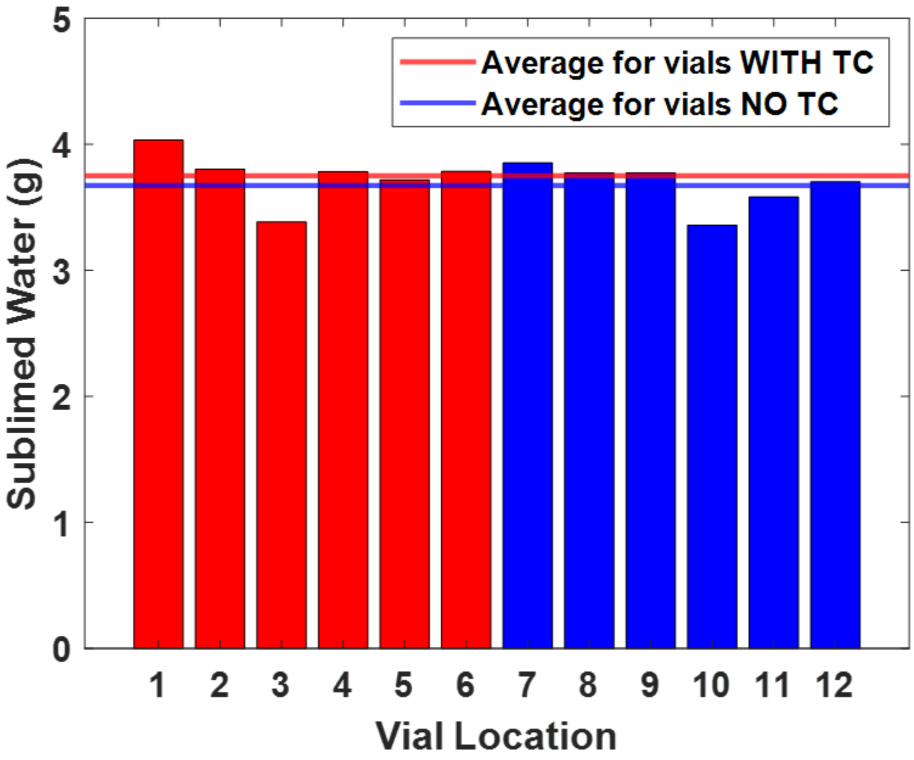

Supplementary Figure 2. The water sublimed from vials with (1-6) and without conventional TCs (7-12).

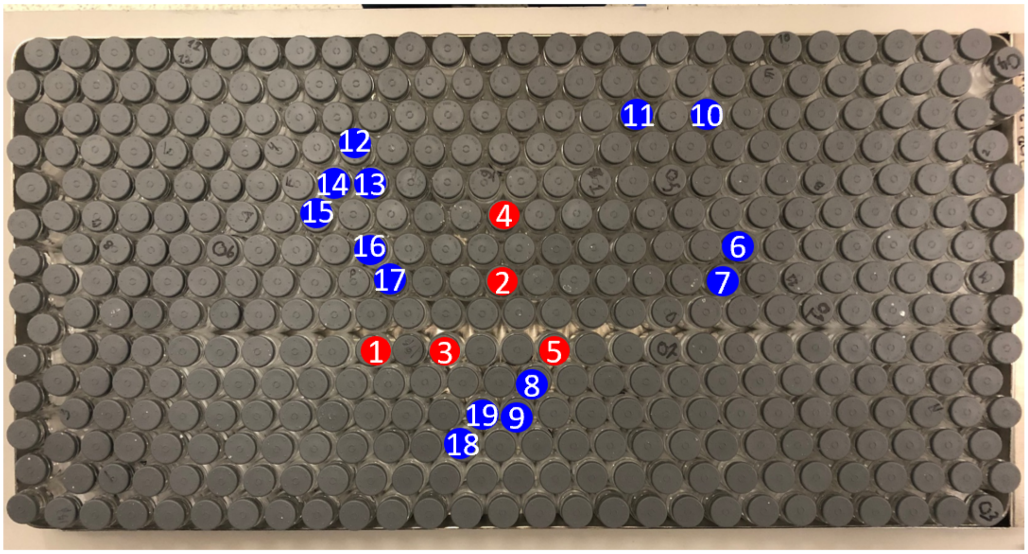

Supplementary Figure 3. The photo of the REVO Millrock shelf with vials: 6R SCHOTT Vials filled with 4 ml 5% mannitol solution. The 19 investigated vials are color-marked.

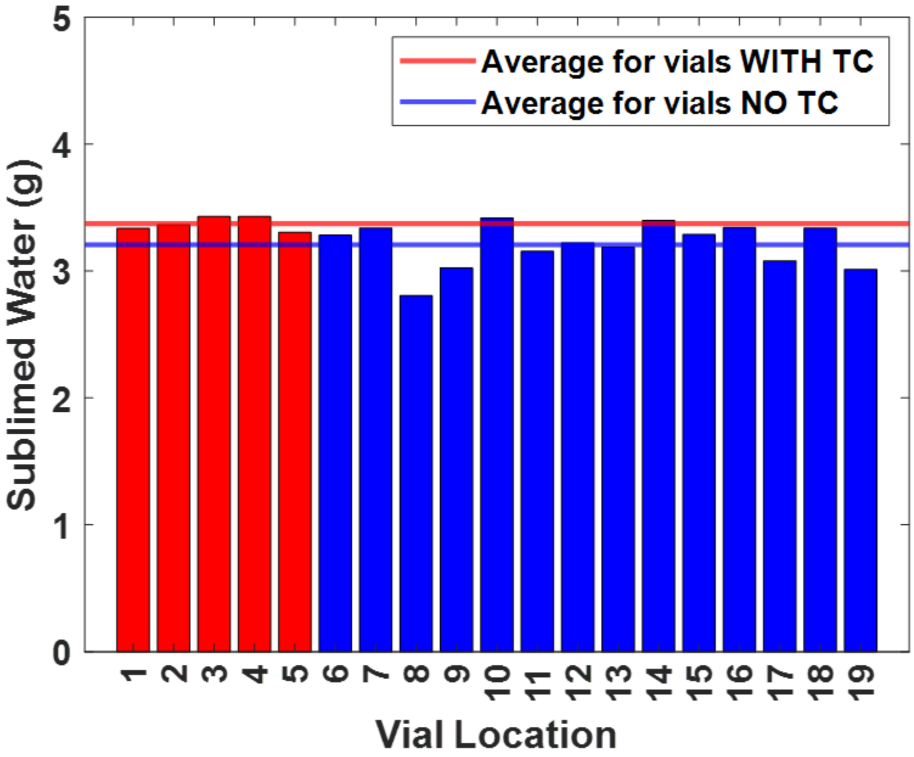

Supplementary Figure 4. The water sublimed from vials with (1-5) and without conventional TCs (6-19).
